# A Family History of Diabetes Modifies the Association between Elevated Urine Albumin Concentration and Hyperglycemia in Nondiabetic Mexican Adolescents

**DOI:** 10.1155/2015/437079

**Published:** 2015-08-11

**Authors:** Aida Jiménez-Corona, Antonio Ávila-Hermosillo, Robert G. Nelson, Guadalupe Ramírez-López

**Affiliations:** ^1^General Directorate of Epidemiology, Health Secretariat, Francisco P Miranda 177, Colonia Lomas de Plateros, Delegación Álvaro Obregón, 01480 Mexico City, DF, Mexico; ^2^Ocular Epidemiology Department, Institute of Ophthalmology Foundation Conde of Valenciana, IAP, Chimalpopoca 14, Colonia Obrera, Delegación Cuauhtémoc, 06800 Mexico City, DF, Mexico; ^3^Regional General Hospital No. 89, Mexican Institute of Social Security, Washington 1988, Colonia Moderna, Sector Juárez, 44150 Guadalajara, JAL, Mexico; ^4^Diabetes Epidemiology and Clinical Research Section, National Institute of Diabetes and Digestive and Kidney Diseases, Phoenix Epidemiology and Clinical Research Branch, 1550 East Indian School Road, Phoenix, AZ 85014, USA; ^5^Adolescent Epidemiological and Health Services Research Unit, Mexican Institute of Social Security, Avenida Tonalá 121, 45400 Guadalajara, JAL, Mexico

## Abstract

We examined the frequency of elevated urine albumin concentration (UAC) and its association with metabolic syndrome (MetS) and metabolic markers in 515 nondiabetic Mexican adolescents stratified by family history of diabetes (FHD). UAC was measured in a first morning urine sample and considered elevated when excretion was ≥20 mg/mL. MetS was defined using International Diabetes Federation criteria. Fasting insulin, insulin resistance, and lipids were evaluated. Multivariate logistic regression was performed. Elevated UAC was present in 12.4% and MetS was present in 8.9% of the adolescents. No association was found between elevated UAC and MetS. Among adolescents with FHD, 18.4% were overweight and 20.7% were obese, whereas, among those without a FHD, 15.9% were overweight and 7.5% were obese. Hyperglycemia was higher in those with elevated UAC than in those without (44.4% versus 5.1%, *p* = 0.003). Hyperglycemia (OR = 9.8, 95% CI 1.6–59.4) and number of MetS components (OR = 4.5, 95% CI 1.5–13.3) were independently associated with elevated UAC. Among female participants, abdominal obesity was associated with elevated UAC (OR = 4.5, 95% CI 1.2–16.9). *Conclusion*. Elevated UAC was associated neither with MetS nor with any metabolic markers in nondiabetic adolescents. However, FHD modified the association of elevated UAC with hyperglycemia and the number of MetS components.

## 1. Introduction

Microalbuminuria is a marker of systemic endothelial dysfunction [1]. In the general population, it predicts multiple outcomes, including type 2 diabetes, hypertension, chronic kidney and cardiovascular diseases, metabolic syndrome (MetS), and all-cause, cardiovascular, and renal mortality [2–5]. During puberty, microalbuminuria may be present as a consequence of changes in renal hemodynamics, insulin resistance, sexual hormones, somatic growth, and the renin-angiotensin-aldosterone system [6].

The prevalence of microalbuminuria varies with age in nondiabetics, ranging from 6.5% to 8.9% in children and adolescents [7, 8], from 4.4% to 5.9% in young adults [9, 10], and from 7.7% to 22.0% in older adults [9]. The prevalence of MetS is between 4.5% and 6.7% in adolescents, depending on the criteria used [11, 12], and it is higher in Mexican-Americans and Mexicans (10.4% to 19.6%) than in non-Hispanic whites [12, 13] and in the presence of obesity (32.8%) [11].

According to the WHO criteria, microalbuminuria is part of the MetS [14], although no other definitions of MetS include it [11–13, 15]. Hypertension, high glucose, high triglycerides, and abdominal obesity are related to microalbuminuria in adults [4, 16], but these relationships are not clear in adolescents. Some investigators have found no association between MetS and microalbuminuria in adolescents [8, 17], whereas others have reported an association among obese adolescents [18, 19].

We examined the frequency of elevated urine albumin concentration (UAC) in nondiabetic Mexican adolescents and its association with MetS and metabolic markers. We also examined these associations after stratifying by the family history of diabetes, since having a first-degree relative with diabetes appears to increase the risk of elevated albuminuria [20].

## 2. Materials and Methods

### 2.1. Study Population

A cross-sectional study was conducted in public high-schools in the suburbs of Guadalajara, Jalisco, Mexico. Study participants included 15–19-year-old adolescents who consented to participate and reported that they were unaware of having any chronic disease. Of the 654 adolescents enrolled in the first year of high-school, 570 (87.2%) agreed to participate in the study. Fifty-five were excluded from the study because of missing data (anthropometric measurements or blood or urine samples). There were no differences between participants and nonparticipants for age (mean = 16 years in both groups), body mass index (BMI) (21.8 versus 21.6 kg/m^2^, *p* = 0.758), menstruation status (98.4% versus 97.6%, *p* = 0.537), or smoking (5.4% versus 9.5%, *p* = 0.183). More boys than girls participated in the study (51.0% versus 49.0%, *p* = 0.016). The protocol was approved by the Institutional Review Board of the Mexican Institute of Social Security and designed based on the Declaration of Helsinki. Written informed assent/consent was obtained from all of the students and their parents.

### 2.2. Measurements

Data (questionnaires and anthropometric and blood pressure measurements) were collected at the schools by trained nutritionists, using standardized procedures. Height, weight, waist circumference, and triceps and subcapsular skin-fold thickness were measured by the Lohman method [21]. Blood pressure was measured using a validated digital Baumanometer (Omron HEM–751; Vernon Hills, IL, USA), after the participant was seated for 5 min, and the average of two measurements was obtained.

A blood sample was obtained after a 12-hour fast by venipuncture and frozen at −80°C until analyzed. Serum glucose concentration was measured using a hexokinase automated method (Synchron CX4, Beckman Coulter Inc., Brea, CA, USA). Serum insulin concentration was measured using an IMMULITE 2000 analyzer (Diagnostic Products Co., Los Angeles, CA, USA) and a solid-phase, two-site chemiluminescent immunometric assay. Insulin resistance was evaluated using the homeostatic model assessment index of insulin resistance (HOMA-IR) calculated as

(1) $$\begin{array}{c} \frac{\text{fasting insulin }\left(\mu U/mL\right)\times \text{fasting glucose }\left(mmol/L\right)}{22.5}. \end{array}$$

Serum triglyceride concentrations were determined using conventional enzymatic procedures. Total serum cholesterol, low-density lipoprotein-cholesterol (LDL-C), and high-density lipoprotein-cholesterol (HDL-C) concentrations were determined using immunochemical methods and an ILab 300 Plus analyzer (Instrumentation Laboratory Ltd., Birchwood, Warrington, UK).

A single-void first morning urine sample was collected and kept at 2–8°C for less than two weeks, until analysis was performed. Adolescents were asked not to perform exercise the day before samples were taken. In girls, urine samples were collected at least 3 days before or 3 days after menstruation. Urine albumin concentration (UAC) was measured using radioimmunoassay and double antibody albumin (Diagnostic Products Co., Los Angeles, CA, USA). In this competitive radioimmunoassay, ^125^I-labeled albumin competes with albumin in the patient sample for antibody binding sites.

### 2.3. Outcome Variable

Urine albumin concentration was classified as normal if it was <20 mg/mL and elevated when it was ≥20 mg/mL [22].

### 2.4. Independent Variables

MetS was defined according to the International Diabetes Federation criteria. For adolescents who were 10-16 years old, MetS includes abdominal obesity (waist circumference ≥90th percentile for age and sex) and two or more of the following: fasting glucose ≥5.6 mmol/L (100 mg/dL); triglycerides ≥1.7 mmol/L (150 mg/dL); HDL-C <1.3 mmol/L (40 mg/dL); and systolic blood pressure ≥130 mm Hg or diastolic blood pressure ≥85 mm Hg. For adolescents who were more than 16 years old, MetS includes abdominal obesity (waist circumference ≥90 cm for boys and ≥80 cm for girls) and two or more of the following: glucose ≥5.6 mmol/L (100 mg/dL); triglycerides ≥1.7 mmol/L (150 mg/dL); HDL-C <1.0 mmol/L (40 mg/dL) for boys and <1.3 mmol/L (50 mg/dL) for girls; and systolic blood pressure ≥130 mm Hg or diastolic blood pressure ≥85 mm Hg [15].

High total serum cholesterol concentration was defined as ≥5.2 mmol/L (200 mg/dL) and high LDL-C as ≥3.4 mmol/L (130 mg/dL), in accordance with the National Cholesterol Education Program criteria [23]. High insulin concentration was defined as ≥90.3 pmol/L (15.05 *μ*U/mL) and a high HOMA-IR value was defined as ≥3.43 [24].

Overweight and obesity were defined according to the International Obesity Task Force BMI criteria that established BMI cutoffs for age and sex corresponding to values of 25 kg/m^2^ for overweight and 30 kg/m^2^ for obesity at 18 years of age [25]. An estimate of the percent body fat was calculated using sex-specific Slaughter equations for the sum of triceps and subscapular skin-folds (≤35 mm or >35 mm) according to Tanner stages (prepubertal, pubertal, or postpubertal stage) [26].

Family history of type 2 diabetes, which was obtained by a questionnaire delivered to parents, was considered to be positive if at least one of the parents had diabetes.

### 2.5. Covariables

Smoking and physical activity habits were obtained by interview using a standardized questionnaire. Smoking was defined as minimum of one cigarette smoked per day during the past month [27]. A physical activity and inactivity questionnaire validated in Mexican children was used to estimate the total hours per day of physical activity performed during the last month, and summarize responses according to the intensity of exercise (light, moderate, or vigorous) [28]. A semiquantitative food-frequency questionnaire [29] estimated the participant's daily average intakes of energy and macronutrients using the Evaluation System of Nutritional Habits and Nutrient Intake (ESNUT). Pubertal development was evaluated according to the Tanner stages using an autoadministered questionnaire that showed photographs and detailed descriptions for each of the five stages (from prepubertal to complete puberty) [30].

### 2.6. Statistical Analysis

Descriptive analysis included the calculation of means, standard deviations, medians, and percentages. Mean differences were estimated using Student's* t*-test for independent normally distributed samples and using Wilcoxon rank-sum test for median differences when the distributions were skewed. Chi-square tests or Fisher's exact test was used to evaluate percentage differences. Crude and adjusted logistic regression analyses were used to evaluate the association between elevated UAC and MetS (or metabolic components). Multivariate logistic regression models were adjusted for sex (male/female), sexual development (II–IV/V), family history of diabetes (yes/no), smoking (yes/no), percent body fat (%), total physical activity (h/day), and protein intake (g/day). Interactions between family history of diabetes and MetS or metabolic markers were evaluated. Subsequently, unadjusted and adjusted logistic regression models were also evaluated after stratifying by family history of diabetes. A Hosmer–Lemeshow test grouped according to deciles of risk was used to evaluate goodness of fit. Stata 9.0 (Stata Corporation, TX) was used for statistical analysis and *p* < 0.05 was considered to be statistically significant.

## 3. Results

A total of 515 nondiabetic adolescents participated in the study. The prevalence of elevated UAC in this cohort was 12.4%. In adolescents with elevated UAC (≥20 mg/mL), the UAC median (percentiles 25–75) was 27.5 mg/mL (23.3–38.0 mg/mL), and, in those without elevated UAC (<20.0 mg/mL), the UAC median was 6.4 mg/mL (3.2–10.0 mg/mL). The prevalence of MetS was 8.9%. Among the participants, 31.7% had no MetS components (including abdominal obesity), 45.1% had one component, 13.4% had two components, and 9.9% had three or more components. Low HDL-C concentration was present in 58.5% of the participants, abdominal obesity in 15.3%, high triglyceride concentrations in 14.0%, high serum glucose concentration in 9.5%, and high blood pressure in 7.8%. The prevalence of high total serum cholesterol concentration was 7.6%, high LDL-C concentration was 10.1%, high fasting insulin concentration was 15.8%, and high insulin resistance was 13.8%.

The sociodemographic and clinical characteristics according to UAC group are shown in Table 1. Waist circumference and percent body fat were lower in adolescents with elevated UAC than in those without elevated UAC (waist circumference = 72.0 ± 7.6 cm versus 75.6 ± 10.4 cm, *p* = 0.021; percent body fat = 26.5 ± 9.9% versus 29.9 ± 11.9%, *p* = 0.026). Although the frequency of hyperglycemia was higher in adolescents with elevated UAC than in those with normal UAC (12.5% versus 9.1%, *p* = 0.384), this difference was not statistically significant. None of the other variables differed between UAC groups.

The relationships between elevated UAC and MetS components and other metabolic markers were examined according to the presence or absence of a family history of diabetes (Table 2). Among adolescents without a family history of diabetes (*n* = 422), waist circumference remained lower in the group with elevated UAC than in the normal UAC group (71.0 ± 6.9 cm versus 74.9 ± 10.3 cm, *p* = 0.013), but the difference in percent body fat, although numerically similar, was no longer statistically significant (26.4 ± 8.9% versus 28.9 ± 11.1%, *p* = 0.120). Among adolescents with a family history of diabetes (*n* = 87), the proportion with high serum glucose concentration was higher in the group with elevated UAC than in the normal UAC group (44.4% versus 5.1%, *p* = 0.003). Neither MetS nor other metabolic markers differed according to UAC group in those with or without a family history of diabetes.

Elevated UAC in the entire cohort was unrelated to MetS or any metabolic markers after adjustment for sex, level of sexual development, smoking, physical activity, protein intake, percent body fat, or family history of diabetes by logistic regression analysis (Table 3). Similar results were found when analyses were performed separately in adolescents who were obese and in those who were not (Table 4).

A strong interaction was found, however, between family history of diabetes and hyperglycemia (*p* = 0.003), so multivariate logistic regression analyses were performed after stratifying by family history of diabetes (Table 5). Among adolescents with a family history of diabetes, hyperglycemia (OR = 9.81, 95% CI 1.62–59.43), and the number of MetS components (OR = 4.48, 95% CI 1.51–13.32) were independently associated with elevated UAC in comparison to normal UAC after controlling for covariates. No other statistically significant associations were found according to family history of diabetes. As no males had abdominal obesity along with elevated UAC, the association between these two parameters was only evaluated in females and was statistically significant (OR = 4.5, 95% CI 1.2–16.9).

## 4. Discussion

In nondiabetic Mexican adolescents, elevated UAC was not associated with MetS or with metabolic markers. These findings are consistent with other studies in adolescents [8, 17], but they contradict findings in obese nondiabetic children [19]. Inconsistencies between studies may be due, in part, to the choice of MetS criteria used in the studies or due to other differences in the populations. Peralta et al. [31], for example, found that among Hispanics residing in the United States, Native American ancestry was associated with a higher prevalence of elevated albuminuria than European ancestry. Additionally, they found that the prevalence of elevated albuminuria was attenuated by higher socioeconomic status. The prevalence of elevated UAC in our study was similar to albuminuria found in the Mexican-American adults in their study.

Microalbuminuria in nondiabetic adolescents and adults has previously been associated with hyperinsulinemia, glucose intolerance, hypertension, or hypercholesterolemia [8, 17, 18, 32]. We report for the first time that fasting hyperglycemia and the number of MetS components are associated with elevated UAC in nondiabetic Mexican adolescents, but only among those with a family history of diabetes. This finding is consistent with the observations from the Mexico City Diabetes Study, in which microalbuminuria was associated with parental history of diabetes and impaired glucose tolerance in nondiabetic Mexican adults [33].

Persons with elevated albuminuria and impaired glucose tolerance are at increased risk for developing type 2 diabetes [5, 33–35]. In the Framingham study, an association between microalbuminuria and hyperglycemia was found 24 years before the diagnosis of type 2 diabetes [34], and the prevalence of microalbuminuria increased with the increasing glycemia even before the onset of diabetes [35]. Furthermore, in adults, family history of diabetes has been recognized as an independent risk factor for type 2 diabetes [36, 37]. In children and adolescents from the Bogalusa Heart Study, parental history of diabetes was the most important risk factor to predict type 2 diabetes (hazard ratio = 2.67, 95% CI 1.58–4.53) [38] and in the TODAY study almost 90% of diabetic American adolescents had a family history of diabetes (including nuclear family and grandparents) [39]. The association of elevated UAC with fasting hyperglycemia among adolescents with a family history of diabetes in the present study suggests that these adolescents are at greater risk for diabetes later in life.

We found that abdominal obesity was associated with elevated UAC only among female adolescents in the total population. In nondiabetic adults, abdominal obesity has been found to be an independent risk factor for microalbuminuria [4], although this was not observed in the Mexico City Diabetes Study [33] and other studies in Venezuelan adults [40]. On the other hand, obesity was independently associated with microalbuminuria in nondiabetic South Asian adults with type 2 diabetic relatives [41]. The same inconsistencies have been observed in adolescents; Hungarians showed a higher urinary albumin/creatinine ratio among obese versus nonobese; however, the difference was not controlled by confounders [18]. On the other hand, in a national sample of adolescents from the United States, the prevalence of microalbuminuria was lower among obese than nonobese [8]. Also, BMI and % body fat were not different by microalbuminuria status in obese children and adolescents [17]. The mechanisms that might explain a relationship between abdominal obesity and microalbuminuria are the presence of low-grade inflammation and endothelial dysfunction [42].

The strengths of our study include measuring UAC by radioimmunoassay in first morning urine samples, which eliminates the postural effects on albumin excretion. Participants were advised to limit physical activity the day before sample collection to reduce the likelihood of exercise-associated albuminuria, and sample collection in the females was done when they were not menstruating. Tanner staging was performed, and data were collected on dietary intake, smoking habits, and level of physical activity, so we could control for these potentially confounding variables. A limitation is that we did not measure the urine creatinine concentration and therefore could not correct for differences in urine volume. Hence, our measurement of UAC does not reflect the current standard for measurement of albuminuria, which is the albumin-to-creatinine ratio in the first morning urine sample [43]. Nevertheless, UAC is reported to have a sensitivity of 88.6% and a specificity of 88.9% for correctly classifying elevated albuminuria when compared with the 24 h urinary albumin excretion rate [44]. In addition, we performed only a single measurement of urinary albumin, which reduces precision relative to multiple measurements made over several days, thereby increasing the chance of misclassifying study participants [45].

## 5. Conclusions

We did not find an association between elevated UAC and MetS in nondiabetic Mexican adolescents. However, fasting hyperglycemia and the number of MetS components were associated with elevated UAC in those with a positive family history of diabetes. Also, female adolescents with abdominal obesity had elevated UAC. All these findings may reflect an increased risk for type 2 diabetes.

## Figures and Tables

**Table 1:** Sociodemographic and clinical characteristics by urine albumin concentration group.

Sociodemographic and clinical characteristic		Normal UAC (*n* = 451)	Elevated UAC (*n* = 64)	*p* value^‡^
Female (%)		47.7	56.3	0.199

Age (years)		16.6 ± 0.9	16.6 ± 1.0	0.795
		16.0 (16.0, 17.0)	16.5 (16.0, 17.0)	

Tanner sexual stages (%)	II–IV	66.5	78.1	0.063
	V	33.5	21.9	

Smoking (%)		5.8	3.2	0.559

Family history of diabetes (%)		12.8	10.3	0.527

Physical activity (h/day)		2.2 ± 1.0	2.2 ± 1.0	0.934
		2.0 (1.5, 2.8)	2.1 (1.4, 2.8)	

Protein intake (g/day)		87.2 ± 36.1	85.5 ± 28.8	0.909
		80.3 (64.5, 100.3)	78.8 (62.8, 105.5)	

Body mass index (kg/m^2^)		22.8 ± 4.4	21.8 ± 3.7	0.057
		22.0 (19.7, 24.9)	20.9 (19.4, 23.0)	

Nutritional status (%)	Low weight	8.0	9.4	0.479
	Normal weight	65.0	71.9	
	Overweight	16.9	14.1	
	Obese	10.2	4.7	

Waist circumference (cm)		75.6 ± 10.4	72.0 ± 7.6	0.021
		73.4 (68.3, 80.7)	71.0 (66.7, 76.1)	

Percent body fat (%)		29.8 ± 11.4	26.5 ± 9.9	0.026
		29.9 (28.5, 30.9)	25.9 (21.9, 30.0)	

Abdominal obesity (%)		16.0	10.9	0.296

Fasting glucose (mmol/L)		4.91 ± 6.43	4.93 ± 0.42	0.597
		4.88 (4.55, 5.27)	4.88 (4.61, 5.27)	

High glucose (%)		9.1	12.5	0.384

Fasting insulin (pmol/L)		64.2 ± 31.8	57.0 ± 22.8	0.132
		56.4 (43.2, 76.8)	49.8 (41.4, 69.0)	

High insulin (%)		16.7	9.4	0.134

HOMA-IR^a^		2.3 ± 1.3	2.1 ± 0.9	0.199
		2.0 (1.5, 2.9)	1.9 (1.5, 2.6)	

High HOMA-IR (%)		14.4	9.4	0.271

Systolic blood pressure (mm Hg)		113.1 ± 9.9	111.4 ± 12.0	0.237
		112.5 (105.5, 120.0)	109.8 (104.3, 119.5)	

Diastolic blood pressure (mm Hg)		68.9 ± 8.7	67.6 ± 7.7	0.261
		68.5 (63.0, 74.5)	67.0 (62.3, 72.9)	

High blood pressure (%)		8.0	6.3	0.628

Triglycerides (mmol/L)		1.3 ± 0.8	1.2 ± 0.6	0.476
		1.1 (0.8, 1.5)	1.0 (0.8, 1.3)	

High triglycerides (%)		14.6	9.4	0.256

Total cholesterol (mmol/L)		4.0 ± 0.9	3.8 ± 0.7	0.137
		3.9 (3.4, 4.5)	3.8 (3.3, 4.3)	

High total cholesterol (%)		8.0	4.7	0.455

HDL-C (mmol/L)		1.1 ± 0.2	1.1 ± 0.2	0.957
		1.1 (0.9, 1.2)	1.1 (0.9, 1.2)	

Low HDL-C (%)		57.4	65.6	0.213

LDL-C (mmol/L)		2.5 ± 0.7	2.4 ± 0.6	0.330
		2.5 (2.1, 2.9)	2.4 (2.0, 2.8)	

High LDL-C (%)		10.2	9.4	0.838

MetS (%)		9.3	6.3	0.638

Number of MetS components^b^ (%)	0	32.6	25.0	0.408
	1	43.7	54.7	
	2	13.5	12.5	
	3	10.2	7.8	

Values are means ± standard deviation, medians (25th percentile, 75th percentile), or percentage.

HDL-C: high-density lipoprotein-cholesterol, HOMA-IR: insulin resistance, LDL-C: low-density lipoprotein-cholesterol, MetS: metabolic syndrome, and UAC: urine albumin concentration.

^‡^Chi square test, Fisher's exact test, Student's *t*-test, or Wilcoxon rank sum test.

^a^HOMA-IR: fasting insulin (*μ*U/mL) × fasting glucose (mmol/L)/22.5.

^b^Including abdominal obesity.

**Table 2:** Metabolic syndrome and metabolic markers by urine albumin concentration group, stratified according to family history of diabetes.

Metabolic syndrome and metabolic markers	Normal UAC	Elevated UAC	*p* value^‡^
	(*n* = 451) %	(*n* = 64) %	
Without family history of diabetes (*n* = 422)			
Waist circumference (cm, mean ± SD)	74.9 ± 10.3	71.0 ± 6.9	0.013
Percent body fat (%, mean ± SD)	28.9 ± 11.1	26.4 ± 8.9	0.120
Obesity	7.9	3.7	0.403
Abdominal obesity	13.9	5.6	0.124
High glucose	9.8	7.4	0.803
High insulin	15.3	7.4	0.147
High HOMA-IR^a^	13.4	7.4	0.275
High blood pressure	8.2	7.4	1.000
High triglycerides	13.6	7.4	0.276
High total cholesterol	7.1	3.7	0.558
Low HDL-C	57.7	63.0	0.456
High LDL-C	8.7	7.4	1.000
MetS	7.9	3.7	0.403
Number of MetS components^b^, (mean ± SD)	1.03 ± 1.03	0.91 ± 0.76	0.730
With family history of diabetes (*n* = 87)			
Waist circumference (cm, mean ± SD)	79.2 ± 10.1	76.8 ± 9.4	0.504
Percent body fat (%, mean ± SD)	34.2 ± 11.6	27.3 ± 15.6	0.225
Obesity	21.8	11.1	0.678
Abdominal obesity	26.9	44.4	0.272
High glucose	5.1	44.4	0.003
High insulin	23.1	11.1	0.411
High HOMA-IR^a^	19.2	11.1	1.000
High blood pressure	7.7	0.0	1.000
High triglycerides	20.5	22.2	1.000
High total cholesterol	11.5	11.1	1.000
Low HDL-C	57.7	77.8	0.304
High LDL-C	16.7	11.1	1.000
MetS	16.7	22.2	0.650
Number of MetS components^b^, (mean ± SD)	1.18 ± 1.09	1.89 ± 1.17	0.069

HDL-C: high-density lipoprotein-cholesterol, HOMA-IR: insulin resistance, LDL-C: low-density lipoprotein-cholesterol, MetS: metabolic syndrome, and UAC: urine albumin concentration.

^‡^Chi square test, Fisher's exact test, Student's *t*-test, or Wilcoxon rank sum test.

^a^HOMA-IR = fasting insulin (*μ*U/mL) × fasting glucose (mmol/L)/22.5.

^b^Including abdominal obesity.

**Table 3:** Crude and adjusted logistic regression analysis between elevated urine albumin concentration and metabolic syndrome and metabolic markers.

Metabolic syndrome and metabolic markers	OR_crude_	95% CI	OR_adjusted_	95% CI
Abdominal obesity	0.65	0.28–1.47	2.13	0.71–6.43
High glucose	1.43	0.64–3.20	1.86	0.80–4.33
High insulin	0.52	0.22–1.24	0.72	0.26–2.03
High HOMA-IR	0.61	0.25–1.48	0.98	0.34–2.84
High blood pressure	0.77	0.26–2.24	1.50	0.47–4.74
High triglycerides	0.60	0.25–1.46	0.89	0.35–2.26
High total cholesterol	0.57	0.17–1.90	0.78	0.22–2.75
Low HDL-C	1.42	0.82–2.45	1.58	0.88–2.84
High LDL-C	0.91	0.37–2.23	1.00	0.36–2.75
MetS	0.65	0.22–1.86	1.97	0.55–7.10
Number of MetS components^a^	1.00	0.77–1.29	1.39	1.00–1.95

HDL-C: high-density lipoprotein-cholesterol, HOMA-IR: insulin resistance, LDL-C: low-density lipoprotein-cholesterol, MetS: metabolic syndrome, and OR: odds ratio.

Models were adjusted by sex, sexual development, smoking, physical activity, protein intake, percent body fat, and family history of diabetes.

^a^Including abdominal obesity.

**Table 4:** Associations between elevated urine albumin concentration and metabolic syndrome or metabolic markers, stratified according to obesity.

Models	OR_crude_	95% CI	OR_adjusted_	95% CI
Without obesity				
Abdominal obesity	1.12	0.42–3.00	1.19	0.43–3.26
High glucose	1.75	0.73–4.19	1.87	0.76–4.57
High insulin	0.75	0.28–1.97	0.60	0.21–1.78
High HOMA-IR	0.76	0.26–2.23	0.59	0.11–2.02
High blood pressure	0.79	0.23–2.69	1.05	0.29–3.79
High triglycerides	0.71	0.27–1.88	0.75	0.28–1.98
High total cholesterol	0.49	0.11–2.14	0.50	0.11–2.22
Low HDL-C	1.52	0.87–2.66	1.42	0.79–2.56
High LDL-C	0.77	0.26–2.24	0.56	0.16–1.93
MetS	1.85	0.50–6.84	2.08	0.55–7.85
Number of MetS components^a^	1.22	0.85–1.75	1.23	0.84–1.79
With obesity				
Abdominal obesity	0.19	0.01–2.59	0.17	0.01–3.34
High glucose	1.27	0.11–15.23	1.54	0.08–30.06
High insulin	0.22	0.02–2.62	0.06	0.001–3.42
High HOMA-IR	0.97	0.08–11.54	0.78	0.05–12.70
High blood pressure	1.59	0.13–19.27	1.28	0.07–21.97
High triglycerides	0.60	0.05–7.03	0.38	0.02–7.47
High total cholesterol	1.80	0.15–21.94	1.47	0.10–20.82
Low HDL-C^b^	—	—	—	—
High LDL-C	5.67	0.47–68.28	4.50	0.30–67.36
MetS	0.24	0.02–2.88	0.05	0.001–2.48
Number of MetS components^a^	1.17	0.32–4.15	0.99	0.20–4.86

HDL-C: high-density lipoprotein-cholesterol, HOMA-IR: insulin resistance, LDL-C: low-density lipoprotein-cholesterol, MetS: metabolic syndrome, and OR: odds ratio.

Models were adjusted by sex, sexual development, smoking, physical activity, protein intake and history of type 2 diabetes.

^a^Including abdominal obesity.

^b^OR was not calculated because of the small sample size for one comparison group in these cells.

**Table 5:** Associations between elevated urine albumin concentration and metabolic syndrome or metabolic markers, stratified according to family history of diabetes.

Models	OR_crude_	95% CI	OR_adjusted_	95% CI
Without family history of diabetes				
Abdominal obesity^a^	0.25	0.06–1.08	0.39	0.12–1.32
High glucose^b^	0.58	0.17–1.94	0.83	0.28–2.47
High insulin^c^	0.35	0.10–1.15	0.72	0.23–2.27
High HOMA-IR^c^	0.41	0.12–1.35	0.98	0.30–3.21
High blood pressure^c^	0.96	0.32–2.84	1.87	0.57–6.17
High triglycerides^c^	0.40	0.12–1.32	0.75	0.25–2.26
High total cholesterol^c^	0.26	0.03–1.98	0.69	0.15–3.17
Low HDL-C^c^	1.35	0.73–2.48	1.35	0.72–2.53
High LDL-C^c^	0.65	0.19–2.23	1.21	0.39–3.78
MetS^d^	0.23	0.03–1.75	1.42	0.26–7.78
Number of MetS components^de^	0.88	0.65–1.19	1.16	0.79–1.70
With family history of diabetes				
Abdominal obesity^a^	2.71	0.62–11.85	3.19	0.68–15.03
High glucose^b^	11.10	1.93–63.85^*∗*^	9.81	1.62–59.43^*∗*^
High insulin^c^	0.48	0.05–4.13	0.64	0.06–6.82
High HOMA-IR^c^	0.60	0.07–5.25	0.92	0.08–10.57
High blood pressure^f^	—	—	—	—
High triglycerides^c^	1.29	0.24–7.01	2.01	0.28–14.45
High total cholesterol^c^	1.10	0.12–9.96	1.93	0.18–21.25
Low HDL-C^c^	2.20	0.42–11.59	4.94	0.70–34.78
High LDL-C^c^	0.71	0.08–6.31	0.91	0.09–9.07
MetS^d^	1.67	0.30–9.19	6.32	0.58–68.90
Number of MetS components^de^	1.69	0.94–3.04	4.48	1.51–13.32^*∗∗*^

HDL-C: high-density lipoprotein-cholesterol, HOMA-IR: insulin resistance, LDL-C: low-density lipoprotein-cholesterol, MetS: metabolic syndrome, and OR: odds ratio.

^a^Adjusted by sex, sexual development, smoking, physical activity, and protein intake.

^b^Adjusted by physical activity, protein intake and percent body fat.

^c^Adjusted by sex, sexual development, smoking, physical activity, protein intake and percent body fat.

^d^Adjusted by sex, sexual development, physical activity, protein intake and percent body fat.

^e^Including abdominal obesity.

^f^OR was not calculated because of the small sample size for one comparison group in these cells.

^*∗*^ *p* value < 0.05, ^*∗∗*^ *p* value < 0.
